# Recalibrating disparities in perceived and actual balance abilities in older adults: a mixed-methods evaluation of a novel exergaming intervention

**DOI:** 10.1186/s12984-018-0369-8

**Published:** 2018-03-22

**Authors:** Toby J. Ellmers, Ioannis Th. Paraskevopoulos, A. Mark Williams, William R. Young

**Affiliations:** 1 Department of Clinical Sciences, Brunel University London, London, UK; 2 Institute of Environment, Health and Societies, Brunel University London, London, UK; 3 Department of Computing and Information Systems, University of Greenwich, London, UK; 4 Department of Health, Kinesiology and Recreation, College of Heath, University of Utah, Salt Lake City, USA

**Keywords:** Self-efficacy, Balance confidence, Perceived balance abilities, Exergame intervention

## Abstract

**Background:**

Published reports suggest a disparity between perceived and actual balance abilities, a trait associated with increased fall-risk in older adults. We investigate whether it is possible to ‘recalibrate’ these disparities using a novel gaming intervention.

**Methods:**

We recruited 26 older adults for a 4-week intervention in which they participated in 8-sessions using a novel gaming intervention designed to provide explicit, augmented feedback related to postural control. Measures of perceived balance abilities (Falls Efficacy Scale-International) and actual postural control (limits of stability) were assessed pre- and post-intervention. We used focus groups to elicit the opinions of participants about how the game may have influenced balance abilities and confidence.

**Results:**

A stronger alignment was observed between postural control and perceived balance capabilities post-intervention (i.e., significant correlations between Falls Efficacy Scale-International scores and limits of stability which were not present pre-intervention). Also, significant improvements in measures of postural control were observed, with these improvements confined to the aspects of postural control for which the exergame provided explicit, augmented feedback. Qualitative data revealed that the intervention made participants more “aware” of their balance abilities.

**Conclusions:**

Our results demonstrate that it is possible to recalibrate the perceptions of older adults relating to their balance abilities through a targeted, short-term intervention. We propose that the post-intervention improvements in postural control may have been, in part, the result of this recalibration; with altered perceptions leading to changes in balance performance. Findings support the application of novel interventions aimed at addressing the psychological factors associated with elderly falls.

**Electronic supplementary material:**

The online version of this article (10.1186/s12984-018-0369-8) contains supplementary material, which is available to authorized users.

## Background

Bandura’s [1] Self-Efficacy Theory states that it is our perceived, rather than actual, capabilities which determine the actions we select. However, an accurate awareness of our physical abilities—both our strengths and our weakness—is critical to ensuring that we avoid taking any unnecessary risks when navigating our environment. For example, before deciding which walking path to select—a shorter, ‘risky’ path covered in ice, or a longer, ‘safer’ path—we need to appraise our physical capabilities. However, approximately one-third of older adults misjudge their balance abilities, either over- or under-estimating their physical capabilities [2].

### Disparities between perceived and actual balance abilities

A discrepancy between perceived and actual capabilities will likely lead to these individuals engaging in either unduly cautious or risky behaviours; both of which may increase the likelihood of a fall occurring. For example, a cautious approach will likely lead to activity avoidance, which in turn is associated with a reduction in physical activity and increased risk of falls [3], while an overly risky approach may increase the likelihood that an individual will attempt a task which they are unable to safely complete. As elderly falls are the leading cause of injury, and mortality from injury, in those aged 65 years and older [4], the development of cost-effective methods to reduce falls is an important public health challenge. One potential method for reducing falls may be through addressing the inaccurate perceptions of fall-risk made by elderly people. While physical training is a commonplace method to target falls in the elderly, modifying inappropriate levels of confidence relative to physical ability is both novel and theoretically achievable through employing principles of motor learning, such as augmented feedback.

Fear of falling is negatively associated with numerous markers of wellbeing in older adults, including a restriction in physical activities, social isolation, decreased quality of life and increased fall-risk [2, 3, 5–7]. These attributes have led to the common view that fear of falling is maladaptive and should be reduced through interventions. However, fear of falling may not always be associated with an increased risk of falling, particularly if this fear represents an accurate appraisal of one’s balance abilities. In these cases, this fear may even reduce the risk of falls by encouraging the individual to avoid exposing themselves to unnecessary risk [8]. Therefore, designing interventions to indiscriminately reduce this fear of falling may have a detrimental effect on actual falls. Furthermore, while approximately 10% of the older adults in the sample studied by Delbaere and colleagues [2] were under-confident in relation to their balance abilities, 20% over-estimated their physical capabilities. Therefore, reducing fear of falling in individuals who have either accurate or over-confident perceptions of their balance abilities may even increase the risk of falls by encouraging these individuals to perform tasks beyond their physical capabilities.

As a result, it might be more important for “intervention programmes to help elderly people develop a realistic appraisal of fall risk or improve physical functioning in concert with addressing fear, rather than just reduce fear of falling” ([2], p.1). Yet, the majority of interventions that address fear of falling in older adults are designed to indiscriminately reduce fear (i.e. [9]), rather than attempting to ‘recalibrate’ these individuals’ perceptions of their balance abilities. Therefore, the aim in this present research is to determine if it is possible to recalibrate perceptions amongst older adults of their balance capabilities through a carefully designed, short-term exergaming intervention.

### Exergaming as a means to deliver augmented feedback

Exergaming (portmanteau of “exercise” and “videogaming”) was selected as the most appropriate means through which to deliver this present intervention due to the ease in which accurate augmented feedback relating to task performance can be provided to the participant. Specifically, we utilised the Nintendo Wii Balance Board (WBB; Nintendo, Kyoto, Japan) to deliver this intervention, due to the device’s reliability in monitoring centre of pressure (COP; [10–12]). Used in conjunction with the Nintendo Wii Fit technology, the Wii Balance Board allows users to shift their body movement to control a virtual avatar in a variety of different videogames. Real-time visual and auditory feedback is used to monitor and control COP.

While commercially available exergames may successfully challenge and train aspects of balance associated with fall-risk, and provide relevant augmented feedback on performance, we are unaware of any commercially available exergames specifically designed to do so. Therefore, it was necessary to design a bespoke, novel exergame for this specific purpose. Scientists have demonstrated that it is possible to interface the WBB with virtual-reality software to create bespoke interactive games designed for specific training needs of older adults [13, 14]. We developed a novel game in this manner. This exergame was based on the classic videogame, PONG. In this modified version of the game, individuals had to use their COP, moving in the anterior-posterior (forwards and backwards) plane to move a paddle to intercept a ball. In this game, the same physical task is repeated approximately 15 times a minute (at varying degrees of difficulty), with augmented performance feedback provided after each attempted movement.

Researchers have reported that the provision of performance feedback can have a positive effect on both self-efficacy/balance confidence and task selection. For example, Lamarche, Gionfriddo, Cline, Gammage and Adkin [15] found that younger adults who received positive feedback (i.e., feedback which praised their performance on a balance task) reported greater levels of balance confidence and subsequently selected more challenging balance tasks, while those who received negative feedback demonstrated task selection evident of reduced risk-taking. It, therefore, seems logical to assume that: (1) under-confident individuals with excessive levels of fear who receive feedback that their balance abilities are better than previously assumed will experience increases in balance confidence (moving their perceived balance abilities to better align with their actual capabilities), while; (2) over-confident individuals who under-estimate their physiological fall-risk and receive feedback that their balance abilities are worse than previously assumed will experience decreases in balance confidence (once again moving their perceived balance abilities to better align with their actual capabilities). However, given difficulties associated with categorising individuals as either under/over-confident (i.e., problems related to utilising a median/dichotomous split to classify individuals as having either low/high levels of balance confidence and physical functioning), we deemed it more appropriate to correlate perceived and actual balance abilities to determine the degree to which these align.

A recent meta-analysis presented by Lesinski, Hortobágyi, Muehlbauer, Gollhofer and Granacher [16] concludes that an “effective BT [balance training] protocol for healthy older adults is characterized by a training period of 11–12 weeks, a training frequency of three sessions per week, a total number of 36–40 training sessions... and a total duration of 91–120 min of BT [balance training] per week” (p. 1737). However, research from the domain of motor learning demonstrates the profound effect that short-term (i.e., single-session) interventions utilising explicit, augmented feedback can have on perceptions of physical capabilities [17–19]; with these altered perceptions persisting following both a 24-h and 1-week retention period following the feedback [19]. Furthermore, Lamarche and colleagues [15] observed altered perceptions about balance in individuals receiving a single piece of performance feedback. Consequently, these findings indicate that it may be possible to target psychological determinants of elderly fall-risk through interventions which are shorter in duration than those needed to target the physical determinants of fall-risk.

Given that the training dosage of the present intervention is substantially shorter than these recommendations (i.e. 8-sessions over a 4-week period), we do not expect (by design) to observe significant improvement in the balance assessments collected. We predict that this short-term intervention will result in psychological, rather than physical, changes. Specifically, we predict that this intervention will provide participants with an environment in which they can critically appraise their balance abilities, resulting in a recalibration of perceptions relating to physical capabilities. This recalibration will be evidenced by a stronger alignment (as represented by stronger correlations) between postural control and perceived balance abilities post-intervention, compared to pre-intervention. We do not expect this recalibration to be evidenced by significant global changes in perceived balance abilities. Instead, we expect to observe changes at both ends of the continuum (i.e., increased confidence in those individuals who were previously under-confident and decreased confidence in those who were previously over-confident). We therefore predicted to observe a significant correlation between pre-intervention perceptions and the change in perceived balance between pre- and post-intervention. This prediction suggests that the largest pre-post intervention increases in balance confidence will be observed in individuals previously reporting the lowest perceptions of their balance abilities. We also predict that this recalibration will also be evident in the qualitative focus group data. While these measures of ‘recalibration’ are the primary outcomes of the present research, the secondary outcomes are an evaluation of any changes in balance performance. While we do not expect to observe any significant post-intervention changes in postural control, if any improvements are observed, these will be specific to the movements performed in the gaming task, for which explicit, augmented feedback is provided (i.e., significant improvements in anterior-posterior measures of postural control, but not those assessing medial-lateral control). If this work is successful in recalibrating perceptions relating to balance abilities in older adults, it may be possible to target aspects of psychological functioning associated with increased fall-risk through carefully-designed, short-term interventions.

## Methods

### Participants

Altogether, 26 older adults (female/male: 21/5; mean ± SD age: 78.1 ± 8.2; mean ± SD Berg Balance Scale [20] scores (possible score range 0–56): 51.1 ± 3.92) were recruited from four sheltered residential accommodation schemes to participate in a 4-week gaming intervention. Gameplay sessions took place in the communal living area within each sheltered accommodation housing scheme and were attended by all participants from that respective scheme (4–7 participants per scheme). The inclusion criteria were: 65 years of age or older; ability to comprehend and complete questionnaires in the English language; ability to comprehend the within-gameplay goals and performance requirements, and; ability to stand unaided for 10 min (5 participants did, however, use a walking aid during daily gait activities). Participants were excluded from participation if they had received a formal diagnosis of any neurological or cognitive disorder, or if they had been prescribed medication specifically for dizziness, vertigo, or vestibular function. One participant reported that they were born outside of the UK, but all participants spoke English as a first language.

### Ethics, consent and permissions

Ethical approval was obtained from the ethics committee at the lead institution and the research was carried out in accordance with the principals laid down by the Declaration of Helsinki. All participants provided informed, written consent.

### Procedure

We employed a within-participant design. Participants completed a 4-week videogame based exercise program, in which they played 2 sessions per-week (8-sessions total) of a novel, replica version of one of the first videogames commercially available: PONG.

#### The present exergame

**Fig. 1:**
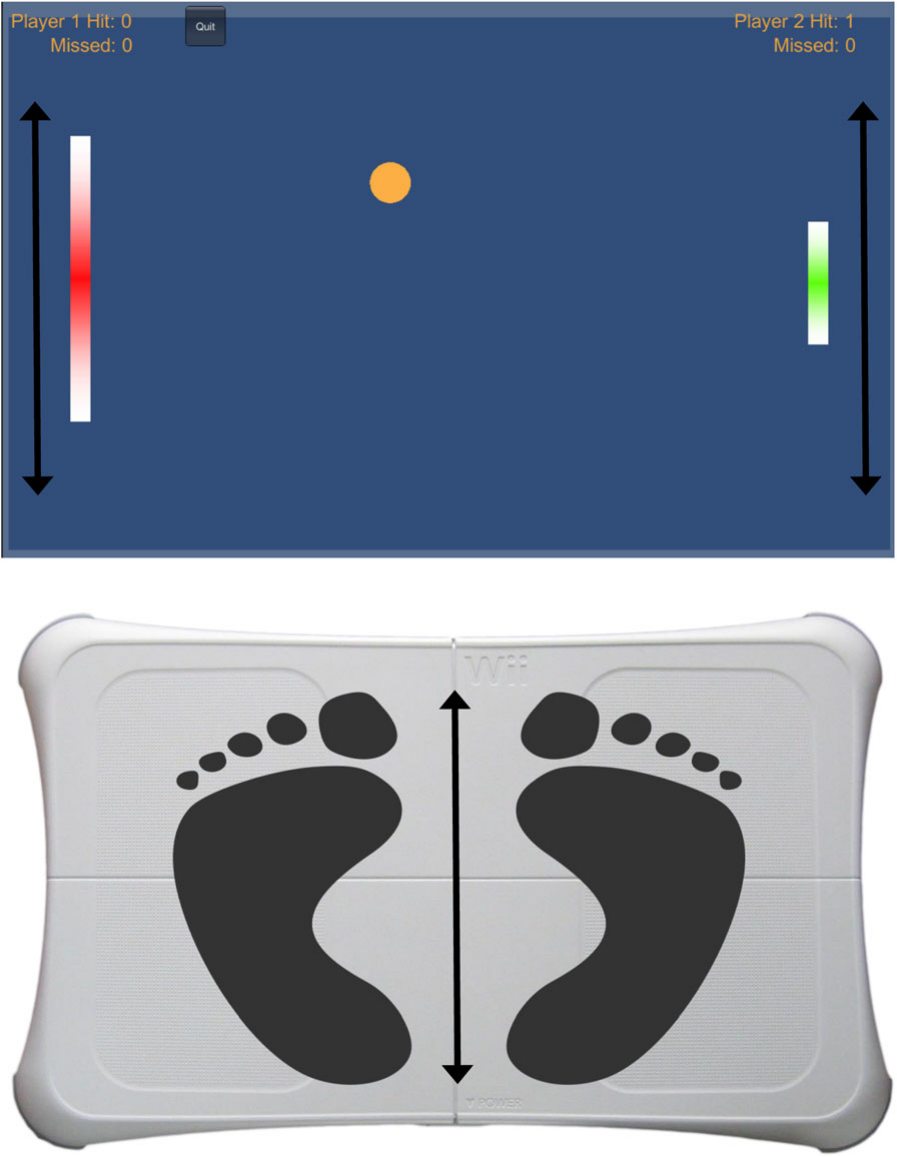
Arrows show the direction in which players will transfer their COP in an attempt to move their paddle to hit the moving ball. Note, the handicapping system is currently being utilised (as indicated by the different paddle sizes); with Player 1 having a Berg Balance Score of 44 and Player 2 having a Berg Balance Score of 56

PONG requires players to move a paddle to intercept a ball (see Fig. 1). In the original PONG game, players moved the paddle by manually rotating a handle. However, by intercepting the Bluetooth signal from a WBB and interfacing it with virtual reality software [13, 14] it became possible to design a novel, replica of the PONG game where players used their balance, moving along the anterior-posterior plane, to control the paddle movement (Fig. 1); moving their COP forwards/anterior direction (to move the paddle up) and backwards/posterior direction (to move the paddle down) to intercept the ball. The game involved participants intercepting the ball in a range of positions up to, including, and sometimes beyond their anterior-posterior limits of stability. Following a familiarisation session (whereby participants attempted approximately 50 ‘hits’ of the ball), participants played 8-sessions of the game spread across a period of 4 weeks, with each participant attempting to ‘hit’ 50 balls per-session (approximately 5 min of gameplay). Research from the domain of motor learning demonstrates altered perceptions of motor capabilities following augmented feedback provided after 30 movement attempts (i.e., [17, 19]), while piloting from the present research and unpublished data from Young et al. [13] suggested that 50-hits represented an exercise duration and intensity that less physically able older adults could still complete. Therefore, 50-hits per gameplay session was selected to ensure both that participants received satisfactory levels of augmented feedback relating to their postural control capabilities, and that the physical demands of the gameplay sessions were appropriate for all levels of physical abilities.

#### Augmented feedback

The game and intervention itself were designed in a manner to maximise the performance feedback provided to participants. As this modified version of PONG presents players with a visual representation of their COP (the paddle) in the anterior-posterior plane, the game provides participants with explicit visual feedback about their postural control abilities (i.e., missing the ball by being unable to successfully move their COP far enough forwards/backwards or successfully moving their COP and hitting the ball). Explicit feedback relating to within-game performance (number of ‘hits’ and ‘misses’) was presented throughout the gameplay session at the top of the screen (Fig. 1). This feedback was further enhanced by participants being able to develop additional awareness of both their balance abilities and limitations through direct indices of postural control performance (i.e., losing stability by leaning too far forwards/backwards and needing the researcher to intervene to help regain stability, or maintaining their balance despite leaning far forwards/backwards). As participants attempted approximately 15 ‘hits’ per minute (at varying degrees of difficulty), with performance feedback (knowledge of whether they hit or missed the ball and of how far forwards/backwards they can move their COP without losing stability) provided after each attempted movement, participants received high frequencies of augmented feedback within a short period of gameplay, albeit relating to specific aspects of their balance (anterior-posterior control and limits of stability).

#### Gameplay difficulty

As recommended in a recent review of the older adult exergaming literature [21], gameplay difficulty was adjusted to each participant’s individual balance abilities. This was achieved by designing an algorithm that adapted individual paddle sizes based on each participant’s scores on the Berg Balance Scale (BBS; [20]); a clinical measure of functional balance. Therefore, higher an individual scored on the BBS (i.e., the higher their functional balance), the smaller their paddle, and so the larger the excursion area across which they would have to move their COP. The mapping of the BBS with the paddle size follows a typical *y* = *ax* + *b* function to accommodate lower paddle size for higher BBS scores, and vice versa. The game engine used to create the game was Unity (United Technologies, San Francisco, USA). Assuming a screen size ratio of 16:10 and a typical paddle size (i.e., the paddle size for the original PONG) within this frame sized at 3 units (Unity game engine units), we map the typical paddle size to the highest BBS for users with maximum independency, which is a BBS of 56 [20]. The lowest BBS score of this range is mapped at a paddle size of 9 units, which is the maximum handicap and allows for a ball of 1 unit size to comfortably get through each side of the paddle, while the paddle is positioned in the middle of the screen. This example handicapping system is then calculated by the following formula:

$$ S=\left(-{0.37}^{\ast }B\right)+24 $$

where *S* = the number of units of adapted paddle size, and *B* = the participant’s BBS score; with a constant within-game ball size of 1 unit (see Fig. 1 for a visual representation of the handicapping procedure).

We implemented this handicapping procedure to ensure that all participants (regardless of their balance abilities) experienced success and failure on the task. We reasoned that this procedure would prevent anyone with either high or low balance abilities experiencing exclusively within-game success or failure, respectively; something which we wanted to prevent, as this performance feedback would likely lead to exclusively positive/negative appraisals of balance ability, regardless of whether this individual was over- or under-confident. However, as gameplay was designed to be challenging, with participants having to move their COP to their limits of stability multiple times per-session, we expected to observe the greatest performance change in those participants with the greatest control over their COP (i.e., the greatest functional balance ability). While initial attempts were made to normalise paddle excursions between different paddle sizes (by applying a ‘gain’ to recorded COP excursions, i.e., increasing the paddle displacement for a given COP displacement), pilot tests revealed that this approach served to increase the difficulty of hitting balls arriving in more central areas (with greater paddle displacements making the paddle more difficult to control). Therefore, paddle excursions were not normalised between paddle sizes.

Gameplay difficulty (paddle sizes, ball speed, location of the shots) was kept constant for each participant throughout the intervention. The intervention was designed to provide participants with clear feedback about their balance abilities and performance; something which changing gameplay difficulty may have obscured. It is likely that the rate/timing of progression would have influenced how participants appraised their balance capabilities. Therefore, while important issues remain regarding task progression/learning in future rehabilitation tools, gameplay difficulty remained constant throughout the current intervention, in order to maintain scientific control over the consistency of the feedback provided. Furthermore, while we anticipated that players would demonstrate improvements in performance within the game, the difficulty of the task was designed so that performance during the final session of gameplay would remain challenging, and still consist of successes and failures.

#### Gameplay protocol

Since intercepting the ball in a position beyond a player’s limits of stability requires a postural adjustment to regain stability, gameplay was supervised by two experimenters who stood directly beside participants to ensure safety. As a result, while loses of stability did frequently occur during gameplay, no participant fell. While all gameplay sessions took place in the presence of the other participants from their respective scheme (4–7 participants per scheme), gameplay was a combination of single- (playing against the computer) and multi-player (playing against a real-life opponent). The physical task, the nature of gameplay and the feedback received relating to postural control capabilities remained identical between single- and multi-player. All participants completed an equal number of single- and multi-player sessions of gameplay; the order of which was counterbalanced across participants. This was to allow all participants sufficient exposure to the two modes of gameplay. This protocol was designed such that, after training, we could explore participants’ perceptions of single- and multi-player gameplay as part of a larger in-depth thematic analysis that is beyond the scope of the current study. Data were excluded from analysis if participants missed any sessions of gameplay. Twenty participants completed all 8-sessions of gameplay.

### Measures

#### Within-game performance

The percentage of ‘hits’ and ‘misses’ from the first and final session of gameplay were recorded. This measure was used to determine the rates at which participants received positive (hits) and negative (misses) feedback, as well as to determine the level of improvement in within-game scores across the intervention.

#### Perceived balance

The Falls Efficacy Scale International (FES-I; [22]) was used to assess perceived balance abilities. The 16-item questionnaire measures the level of concern about falling during a range of activities, both inside and outside of the home. Items are rated on a 4-point Likert scale (1 = *not at all concerned*; 4 = *very concerned*). Scores range from 16 to 64, with higher scores reflecting lower levels of balance confidence. The questionnaire has both excellent internal validity and test-retest reliability [22], and has been previously used to assess perceived balance abilities/perceived physiological fall-risk [2]. This measure was collected pre- and post-intervention.

#### Postural control

The Centre of Pressure Excursion (COPE; [23]) was used to test participants’ postural control capabilities. This test assesses limits of stability, defined as the maximum distance an individual can move their centre of pressure without displacing their base of support. The test involves an individual’s COP being measured while standing on a force plate and leaning as far forward, backward, and sideways (left and right). While the COPE is not a measurement of functional balance, per se, it shares many similarities with clinical measures of functional balance, such as the Functional Reach Test (whereby participants have to lean as far forwards as possible [24]). Separate COPE scores (measured in cm) were calculated for both anterior-posterior (*Y* axis) and medial-lateral (*X* axis) directions. These were recorded on a WBB using the following formula [25]:

$$ X=\frac{L}{2}\kern0.5em \frac{\left(F2+F4\right)-\left(F1+F3\right)}{F} $$

$$ Y=\frac{L}{2}\kern0.5em \frac{\left(F1+F2\right)-\left(F3+F4\right)}{F} $$

where *F*1, *F*2, *F*3, and *F*4 = the ground reaction forces received from the four WBB load cells when under pressure; *L* = the distance (cm) between each load cell (*X* direction = 43.3 cm, *Y* direction = 23.8 cm); *F* = *F*1 + *F*2 + *F*3 + *F*4; and (*X*, *Y*) = coordinates of the COP. Within-participant foot positioning was kept constant for each separate COPE assessment. The WBB has been demonstrated to be an effective method for monitoring COP location, comparable to laboratory-based force plates [11]. COPE scores (cm) were collected pre- and post-intervention, for both the anterior-posterior (*Y* axis) and medial-lateral (*X* axis) directions. Pre-intervention scores were recorded directly prior to the familiarisation gameplay session, while post-intervention scores were recorded following the eighth and final session of gameplay.

### Statistical analysis

We used separate repeated measures ANOVAs compare: (1) the percentage of within-gameplay ‘hits’ during the 8 sessions of gameplay; and (2) the percentage of within-gameplay ‘misses’ during the 8 sessions of gameplay. Bonferonni post-hoc tests were used to follow up any statistically significant results. Effect size is reported as partial eta squared. This analysis was used to determine the rates of both positive and negative feedback the participants received throughout the intervention. We used Pearson’s correlation to assess the relationship between perceived and actual balance abilities. Separate correlations were carried out to compare: (1) Pre-FES-I with pre-anterior-posterior COPE; (2) Pre-FES-I with pre-medial-lateral COPE; (3) Post-FES-I with post-anterior-posterior COPE; and (4) Post-FES-I with post-medial-lateral COPE. Three participants were excluded from any analyses involving FES-I scores, as they were absent from either the pre- or post-gameplay intake session when questionnaires were completed.

We used separate paired-samples *t*-tests to compare pre- and post-intervention scores for: FES-I; anterior-posterior COPE, and; medial-lateral COPE. The effect size is reported as Cohen’s *d.* As we did not predict a significant change in FES-I scores, but instead expected to observe changes on both end of the continuum (i.e., increased confidence in those individuals who were previously under-confident and decreased confidence in those who were previously over-confident), Pearson’s correlation was used to compare the relationship between pre-FES-I scores and the change in FES-I scores between pre- and post-intervention (as represented as the numerical difference between pre- and post-intervention scores).

### Focus groups

Given the complex processes that interact to contribute to one’s perceived balance abilities, we employed focus groups to assess in detail any perceived changes in psychological and physical functioning not detected by the measures collected. The first focus group occurred directly after the fourth session of gameplay, while the second focus group occurred following the eighth and final session of gameplay. We chose to include this mid-point focus group as a means to investigate any ongoing changes in psychological and physical functioning, rather than having to rely solely on the accuracy of participant’s retrospective reports. Each focus group session lasted approximately 30 min. They occurred within the communal area of each of the 4 sheltered accommodation schemes and were attended by each participant from that respective scheme (4–7 participants per-focus group).

Separate topic guides were generated for the two focus groups, which contained both the aims and objectives of each session, as well as a list of open-ended questions. These open ended questions were used to guide the discussions, and to enable participants to discuss their experiences whilst remaining on topic, as is advocated in focus group research [26]. The role of the researcher in the focus groups was to facilitate the discussion; involvement was kept to a minimum aside from asking the questions, providing prompts when necessary and ensuring everyone had an opportunity to contribute. All focus groups were audio recorded and transcribed verbatim.

### Qualitative analysis

Focus group data were analysed through theoretical thematic analysis, as outlined by Braun and Clarke [27]. Following transcription, the lead researcher completed a ‘generation of initial codes’ for each focus group transcript. Analysis was conducted across the whole data set so that full consideration could be given to repeated patterns within the data. Codes were organised into groups through a process of clustering and re-clustering, which allowed relevant themes and sub-themes to develop. As there were similar themes across all datasets, individual focus groups were merged together as one dataset. Broader themes were identified by assessment of commonalities across the dataset, which led to the production of an initial thematic map. The main themes identified were reviewed and refined by the research team at both a micro- and macro-level. Following this stage, themes were examined against the entire data set to ensure that they reflected what the data set conveys as a whole.

## Results

### Quantitative analyses

#### Within-game performance

There was a significant main effect of Gameplay Session on the percentage of within-gameplay ‘hits’ (*F*(7,133) = 5.37, *p* < 0.001, *ƞp*^*2*^ = 0.22). Bonferonni post-hoc tests revealed that when compared to the first session of gameplay, participants displayed a significantly greater percentage of within-gameplay ‘hits’ during: session 4 (*p* = 0.049); session 6 (*p* = 0.012); session 7 (*p* = 0.008), and; session 8 (*p* = 0.001). Participants also displayed a significantly greater percentage of within-gameplay ‘hits’ during the final session of gameplay, compared to the second session (*p* = 0.004).

There was also a significant main effect of Gameplay Session on the percentage of within-gameplay ‘misses’ (*F*(7,133) = 5.37, *p* < 0.001, *ƞp*^*2*^ = 0.22). Bonferonni post-hoc tests also revealed that when compared to the first session of gameplay, participants displayed a significantly greater percentage of within-gameplay ‘misses’ during: session 4 (*p* = 0.049); session 6 (*p* = 0.012); session 7 (*p* = 0.008), and; session 8 (*p* = 0.001). Participants also displayed a significantly greater percentage of within-gameplay ‘hits’ during the final session of gameplay, compared to the second session (*p* = 0.004). These data are presented in Table 1.

**Table 1:** Within-game performance (presented as the percentage of ‘hits’ and ‘misses’) throughout the 8 sessions of gameplay

Gameplay session	Hits mean (± standard deviation)	Misses mean (± standard deviation)
1	58.55% (8.75)	41.45% (8.75)
2	61.79% (10.28)	38.21% (10.28)
3	65.72% (14.21)	34.28% (14.21)
4	68.17% (11.37)^a^	31.83% (11.37)^a^
5	66.27% (11.47)	33.73% (11.47)
6	70.42% (10.75)^a^	29.58% (10.75)^a^
7	70.35% (10.47)^a^	29.65% (10.47)^a^
8	70.77% (7.93)^a, b^	29.23% (7.93)^a, b^

^a^Statistically significant when compared to performance within the first session, ^b^statistically significant when compared to performance within the second session

#### Perceived versus actual balance

There were no significant correlations observed between pre-FES-I scores and pre-intervention scores for either anterior-posterior COPE (*r* = −.29, *p* = 0.13) or medial-lateral COPE (*r* = −.33, *p* = 0.10). There were, however, significant negative correlations observed between post-FES-I scores and both anterior-posterior COPE (*r* = −.49, *p* = 0.02) and medial-lateral COPE (*r* = −.53, *p* = 0.01) scores at post-intervention.

#### Perceived balance

**Fig. 2:**
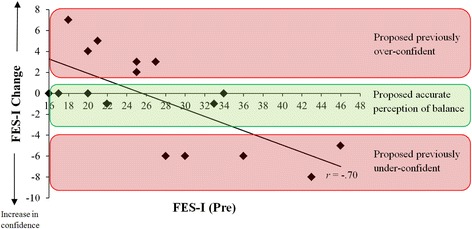
Correlation between pre-FES-I scores and the change in FES-I scores between pre- and post-intervention (as represented as the numerical difference between pre- and post-intervention scores, with a negative change indicating increased balance confidence). The coloured boxes denote the proposed individuals with both inaccurate and accurate perceptions of their balance abilities

A paired-samples *t*-test revealed no significant difference between pre- and post-intervention FES-I scores, *t* (16) = 0.50, *p* = 0.63, *d* = 0.08. These data are presented in Table 2. A significant, negative correlation was observed between pre-FES-I scores and the change in FES-I scores between pre- and post-intervention (as represented as the numerical difference between pre- and post-intervention scores) (*r* = −.70, *p* = 0.001). These data are presented in Fig. 2.

**Table 2:** Mean ± SD values for the assessments collected

	Pre-intervention	Post-intervention
Anterior-Posterior COPE (cm)	15.95 ± 2.88	17.96 ± 2.00***
Medial-Lateral COPE (cm)	25.79 ± 6.08	26.82 ± 4.82
FES-I	27.12 ± 8.88	26.47 ± 6.70

***p < 0.001

#### Postural control

**Fig. 3:**
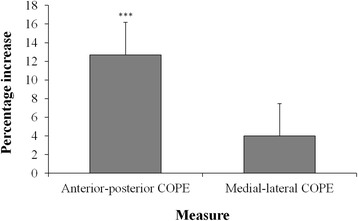
Mean post-intervention increase (mean % increase ± standard error of the mean) in anterior-posterior COPE and medial-lateral COPE. *** post-intervention change significant to *p* < 0.001

A paired-samples *t*-test revealed significantly greater anterior-posterior COPE scores at post-intervention, compared to pre-intervention, *t* (19) = − 4.76, *p* < 0.001, *d* = 0.82. A paired-samples t-test revealed no significant difference between pre- and post-intervention medial-lateral COPE scores, *t* (19) = − 1.23, *p* = 0.12, *d* = 0.19. These data are presented in Fig. 3 and Table 2.

### Qualitative analysis

Analysis of the data collected from focus groups resulted in a single key theme: Awareness of one’s own abilities (which had two sub-themes: Awareness of strengths (leading to increased confidence), and; Awareness of weaknesses (leading to reduced risk-taking)).

In contrast to the lack of significant change in FES-I scores, participants verbally reported increased levels of balance confidence following gameplay. For example, one participant replied: *“As I played it each time... I felt a bit more confident each time, yes”* (female, 76). Comments indicated that these increases in confidence were the result of an improved recognition of one’s abilities. Participants talked about how the game had made them more aware of their perceived strengths in their balance abilities:

*“You’re learning the things you can do rather than the things you can’t do”* (male, 80).

*“I think it makes you realise just how much you can do. You know reaching forwards and like standing up there [playing the game] … I think it makes you realise that”* (female, 82).

Numerous participants suggested that playing the game had made them aware that they could do more things, in relation to their balance, than previously assumed. These changes in confidence appeared to translate directly to behaviour, with participants developing the confidence needed to attempt more challenging balance tasks in daily life. For example, one participant who previously talked about her fears of falling whilst taking a shower commented: “*I’m more confident that I am alright standing to do things. I find showering a lot easier than I used to. I’ve always done with one hand holding on but now I can manage to free stand for a little while”* (female, 85). This apparent relationship between perceived and actual balance was summarised by one participant who stated: “*Once you feel better about it [perceived balance], it helps you with your balance”* (female, 78).

In contrast, other participants reported that the game made them more aware of their balance limitations, with one participant commenting: *“There is definitely a benefit to it [participating in the intervention]… discovering I'm not very good on my toes”* (female, 65). Another participant stated their shock at realising their balance limitations: *“I would have thought I could have done a lot better, but I was surprised”* (female, 72). As a result of this new-found awareness of their limitations, participants reported a reduction in risk-taking, with individuals stating that they no longer attempted to execute movements that could increase their likelihood of falling:

*“Doing this has made me more aware you know, definitely made me more aware. I might have bent down to do something whereas I think now can I do that or am I going to fall over”* (female, 88).

*“One thing it’s taught me is not to try and do something I can’t do. You know like reaching a long way forward. I mean if I can’t reach I have to get a bit nearer before I pick it up”* (male, 80).

## Discussion

### Recalibrating perceptions of balance capabilities

We examined the potential for using a simple, targeted and short-term gaming intervention to reduce discrepancies between perceived and actual balance abilities (shown to be associated with fall-risk; [2, 28]) in older adults. As predicted, we observed a stronger alignment (as represented by a stronger, significant correlation post- rather than pre-intervention; see Section 3.1.2) between actual (as measured by the COPE) and perceived balance abilities (as measured by balance confidence) following 8-sessions of gameplay. This quantitative recalibration was corroborated by data presented from focus groups, where participants reported being more “aware” of their balance abilities; both their strengths and limitations. As a result, it appears as if gameplay promoted an environment in which participants could critically appraise their balance abilities. Individuals who experienced an increase in perceived balance abilities reported greater balance confidence, while reduced risk-taking was reported in those who experienced greater awareness of their balance limitations.

In contrast to the lack of significant changes in FES-I scores (which were utilised to assess perceived balance abilities), a significant correlation was observed between pre-FES-I scores and the change in FES-I scores between pre- and post-intervention (as represented as the numerical difference between pre- and post-intervention scores). A number of participants (11 out of 17) reported changes in FES-I scores of two of more units; however, these changes were characterised by both increases and decreases in balance confidence (see Fig. 2). Specifically, those with low balance confidence (i.e., high FES-I scores) at pre-intervention reported increases in balance confidence, while those with high balance confidence (i.e., low FES-I scores) reported reductions in balance confidence. As there was a stronger alignment between perceived and actual balance abilities post-intervention, we propose that these changes in FES-I scores occurred in those individuals who had previously over/under-estimated their balance abilities (as illustrated in Fig. 2). However, further research is needed to confirm this speculative interpretation.

### Enhanced postural control

We observed significant post-intervention improvements in measures of anterior-posterior postural control (see Fig. 3). This was unexpected, as the intervention utilised in the present research was substantially shorter than the recommendations presented by Lesinski and colleagues [16] with regards to designing balance training protocols for older adults. We suggest that these unexpected findings relate to post-intervention changes in the level of “awareness” of participants’ strengths and limitations, in relation to balance. Previously, researchers have demonstrated the discrepancy between perceived postural capabilities and their actual postural performance levels in older adults [2, 28–31]. It is not known exactly how poor awareness of one’s own action capabilities impacts performance on measures of postural control, although Bandura’s [1] Self-Efficacy Theory proposes that an underestimation of one’s postural capabilities will result in a cautious approach to any balance assessments, with individuals performing below their actual abilities. In contrast to the lack of significant improvements in quantitative measures of balance confidence, a number of participants cited improvements in confidence resulting from these enhanced perceptions of their balance capabilities. Their comments indicated that these altered perceptions subsequently influenced their behaviour, with these individuals feeling more confident to attempt challenging balance tasks in their daily life. We propose that these observed post-intervention improvements in postural control may have been, in part, the result of a recalibration of the participants’ perceptions of their balance capabilities. These altered thoughts and perceptions lead to these previously under-confident individuals realising that they could push themselves further on these assessments and performing closer to their actual limits of physical ability. This finding supports research from other domains, demonstrating enhanced performance on a range of different motor tasks following positive feedback, through the mediating variable of enhanced perceptions of physical capabilities [17, 18].

While significant improvements were observed in postural control, these were confined to anterior-postural COPE, with no significant improvements observed in measures of medial-lateral postural control (as illustrated in Fig. 3). Significant improvements in postural control were, therefore, confined to the aspect of postural control for which gameplay provided direct, augmented feedback. This finding would indicate that while this short-term intervention appears to have helped participants recalibrate their general perceived and actual balance abilities, the improvements observed in measures of postural control may be the result of a task-specific recalibration by virtue of the within-task augmented feedback. Therefore, while a participant may have experienced general increases in their balance confidence as a result of discovering that they could achieve more than previously assumed, we propose that it was the specific within-task feedback relating to their anterior-posterior limits of stability which resulted in the significant improvements observed in measures of anterior-posterior, and not medial-lateral, limits of stability. If these improvements in anterior-posterior postural control were the result of global improvements in psychological or physical functioning, we would have expected to observe significant improvements in aspects of balance for which specific within-task feedback was not provided (i.e., medial-lateral COPE).

Previously, researchers have reported that effective older adult balance training is characterised by: “[lasting] 11–12 weeks, a training frequency of three sessions per week, a total number of 36–40 training sessions... and a total duration of 91–120 min of BT [balance training] per week” ([15], p. 1737). However, our results suggest that it may be possible to induce significant improvements in older adult postural control with carefully-designed, short-term interventions. While we suggested that these improvements may, in part, be mediated by altered appraisals of balance capabilities resulting from augmented feedback received during the intervention, further research is needed to confirm this speculation. For example, as post-intervention improvements in postural control were restricted to what was practiced during the intervention (i.e., anterior-posterior, rather than medial-lateral, postural control), it is possible that similar patterns of results may be observed in older adults participating in challenging balance sessions which do not provide augmented feedback. Therefore, future research should look to replicate these findings utilising a research design that features a control group participating in a balance intervention which does not provide direct augmented performance feedback. Furthermore, without the utilisation of longer-term measures, we are unable to determine if these positive changes following the completion of the present short-term intervention persisted over time.

### Reduced risk-taking

Our data indicate that while some participants voiced higher levels of confidence concerning their balance abilities following gameplay, others became more aware of their limitations; with this increased awareness resulting in a reduction in risk-taking. As a result, the aforementioned reported lack of significant change in quantitative measures of balance confidence is likely caused by previously over-confident (or ‘risky’) participants experiencing decreases in balance confidence as a result of this so-called recalibration, with their balance confidence now more accurately aligned with their physical capabilities.

The game utilised in the present intervention was designed to provide participants with explicit feedback about their performance. If the present intervention was successful in recalibrating the perception of balance capabilities in participants, it is entirely plausible that the intervention may reduce fall-risk through promoting a reduction in risk-taking [28]. This suggestion is further supported by the qualitative data presented where numerous participants reported adopting a more careful strategy when it came to executing potentially risky movements, with other participants indicating that post-intervention they dedicated more attention towards perceiving risks and selecting appropriate movement strategies.

A discrepancy between perceived-and-actual postural ability can lead to the selection of riskier behaviours in older adults [30], with so-called ‘high-risk’ older adults often displaying an over-confident perception of their balance ability [29, 31]. This observation is particularly worrisome, as risk-taking behaviour is an independent risk factor for falls [28]. Given the positive impact that the current intervention may present for future fall-prevention strategies, researchers should try to further establish these links. Many public health organisations list the objective of increasing balance confidence when designing interventions to reduce the risk of falling (i.e., [9]). However, the collective literature suggests that public health interventions may be better served by recalibrating perceptions of balance capabilities, rather than solely aiming to enhance balance confidence.

### Ethical considerations

The potential ethical implications of any future intervention aiming to maximise this recalibration effect must be acknowledged. Presumably, for this recalibration to occur in individuals with over-confident perceptions of their balance abilities, these individuals must be presented with a situation in which their actual capabilities fail to reach their expectations. LaFargue and colleagues [30] suggest that “fall prevention training could be based on exercises in which the elderly explicitly learn to acknowledge their physical limitations” (p. 6). While these lowered perceptions may effectively reduce falls in elderly individuals who had previously displayed risk-taking behaviours, such a strategy is not without concerns ethically. For example, lowered perceptions of balance abilities may, in turn, lead to activity restriction and associated physical deconditioning and a subsequent increase in fall-risk [3]. The ethical dilemmas raised by such a strategy relate to the potential costs and benefits of: (1) attempting to recalibrate ‘over-confident’ individuals’ perceptions of their balance abilities at the risk of the negative consequences of reduced balance confidence, when compared to; (2) keeping ‘over-confident’ individuals active, at the risk of them attempting tasks that are, in their case, unsafe. We argue that the success and the integrity of future strategies will hinge on the ability to recalibrate, rather than indiscriminately reduce, perceptions of balance abilities.

Another ethical consideration relates to the risk of physical injury during the intervention. The losses of stability that, in our estimation, needed to occur in order for these individuals to acknowledge the discrepancy between their perceived and actual balance abilities necessitates that all training must be supervised by a trained physiotherapist/researcher to ensure that no falls occur following these loses of stability. While this recalibration of perceived abilities may be effective in preventing elderly risk-taking and subsequently reducing the rate of falls, the potential psychological (i.e. reduced balance confidence) and physical risk (i.e. falls during the intervention) which may be caused by this method must be taken into consideration and warrants further exploration.

### Limitations

The main limitation of the present research relates to the lack of either a passive or active control group. As a result, one cannot completely exclude the possibility that the observed improvements were simply the result of a learning-effect, with improvements in postural control caused by increased familiarisation with the measures assessed. If our results could be accounted for by this explanation, we would have observed significant improvements in all postural control measures collected. However, post-intervention improvements were only observed in anterior-posterior COPE – aspects of postural control specifically trained with the intervention – with no observed improvements occurring in aspects of postural control not targeted, such as medial-lateral COPE. While we propose that the augmented feedback delivered within the intervention was integral in allowing older adults to develop accurate appraisals of their balance abilities, without a control group, this suggestion remains a speculation. For example, it is possible that similar patterns of results may be observed in older adults participating in challenging balance sessions which do not provide augmented feedback. Consequently, future research should look to replicate these findings utilising a research design that features both a passive control group and an active control group participating in a balance intervention which does not provide direct augmented performance feedback. Another limitation of the present research relates to the relatively small sample size (20 participants) analysed. Whilst this number is above average for both exergaming balance interventions (for example, a recent review reported that 40% of exergaming balance studies included five participants or fewer [32]) and mixed-method research [26], the power of the present study is nonetheless a limitation that must be acknowledged. Finally, without the utilisation of a retention period, we are unable to ascertain if these altered perceptions are maintained without continual training/feedback. While research from the domain of motor learning suggests that altered perceptions of motor capabilities may persist following a no-training/feedback period [19], future research utilising a retention period is needed to confirm these speculations for perceptions relating to postural control.

## Conclusions

Our results demonstrate that it is possible to recalibrate perceptions of balance ability in older adults through a targeted, short-term gaming intervention. This recalibration was represented by a stronger alignment (i.e., a stronger, significant correlation) between actual and perceived balance abilities post-intervention. We also observed significant improvements in measures of postural control. This finding was unexpected, as the intervention developed was substantially shorter than the recommendations for designing training protocols for older adults. Focus group data revealed that the improvements could be a consequence of changes in perceived action capabilities, with participants stating that they were more “aware” of their balance abilities. This increased awareness resulted in a number of participants reporting increased confidence, with these participants developing the confidence needed to attempt more challenging balance tasks in daily life. We propose that post-intervention improvements in postural control may have been the result of a ‘recalibration’ of participants’ perception of their balance capabilities. As these results demonstrate the positive effect that participating in a carefully designed intervention can have on the psychological factors which may mediate balance/fall-risk, these principles of intervention-design (i.e., frequent, augmented feedback) should be taken into consideration in a discipline which focuses predominantly on methods to induce physiological change and improvements in motor control.

## Additional file

Additional file 1: Qualitative Research Check-list. (DOCX 17 kb)
