# Supplementary material for: Recalibrating disparities in perceived and actual balance abilities in older adults: a mixed-methods evaluation of a novel exergaming intervention
Source: J Neuroeng Rehabil. 2018 Mar 22;15:26. doi: 10.1186/s12984-018-0369-8 (PMC5863809; doi:10.1186/s12984-018-0369-8)
Supplement: Supplementary file 1 — Qualitative Research Check-list. (DOCX 17 kb) [file 12984_2018_369_MOESM1_ESM.docx]

**Additional file 1: Qualitative Research Check-list**

| **No** | **Item** | **Guide questions/description** |
| --- | --- | --- |
| **Domain 1: Research team and reflexivity** |  |  |
| Personal Characteristics |  |  |
| 1. | Interviewer/facilitator | Which author/s conducted the interview or focus group? **Author TJE** |
| 2. | Credentials | What were the researcher's credentials? *E.g. PhD, MD* **MSc** |
| 3. | Occupation | What was their occupation at the time of the study? **PhD Student** |
| 4. | Gender | Was the researcher male or female? **Male** |
| 5. | Experience and training | What experience or training did the researcher have? **Author TJE has completed various postgraduate research courses on conducting and analysing qualitative research** |
| Relationship with participants |  |  |
| 6. | Relationship established | Was a relationship established prior to study commencement? **Yes – Author TJE delivered the intervention** |
| 7. | Participant knowledge of the interviewer | What did the participants know about the researcher? e*.g. personal goals, reasons for doing the research* **Participants were aware that the purpose of the qualitative aspect of the study was to investigate any perceived changes in psychological and physiological functioning.** |
| 8. | Interviewer characteristics | What characteristics were reported about the interviewer/facilitator? e.g. *Bias, assumptions, reasons and interests in the research topic* **As the analysis was inductive in nature, no bias were reported.** |
| **Domain 2: study design** |  |  |
| Theoretical framework |  |  |
| 9. | Methodological orientation and Theory | What methodological orientation was stated to underpin the study? *e.g. grounded theory, discourse analysis, ethnography, phenomenology, content analysis* **Thematic analysis** |
| Participant selection |  |  |
| 10. | Sampling | How were participants selected? *e.g. purposive, convenience, consecutive, snowball* **Participants were recruited for the qualitative aspect of the study if they had participated in the balance training intervention** |
| 11. | Method of approach | How were participants approached? e*.g. face-to-face, telephone, mail, email* **Participants were approached face to face during a visit to their sheltered accommodation scheme by authors TJE and WRY** |
| 12. | Sample size | How many participants were in the study? **Twenty-six** |
| 13. | Non-participation | How many people refused to participate or dropped out? Reasons? **Six participants dropped out** |
| Setting |  |  |
| 14. | Setting of data collection | Where was the data collected? e*.g. home, clinic, workplace* **In a common room/residents lounge at the participants’ sheltered accommodation** |
| 15. | Presence of non-participants | Was anyone else present besides the participants and researchers? **No** |
| 16. | Description of sample | What are the important characteristics of the sample? *e.g. demographic data, date* **Important demographic data is reported in the study** |
| Data collection |  |  |
| 17. | Interview guide | Were questions, prompts, guides provided by the authors? Was it pilot tested? **A semi-structured interview guide was used by the researchers. This guide was pilot tested.** |
| 18. | Repeat interviews | Were repeat interviews carried out? If yes, how many? **Participants participated in 3 focus groups.** |
| 19. | Audio/visual recording | Did the research use audio or visual recording to collect the data? **Audio recording** |
| 20. | Field notes | Were field notes made during and/or after the interview or focus group? **No** |
| 21. | Duration | What was the duration of the interviews or focus group? **Approx 30-minutes** |
| 22. | Data saturation | Was data saturation discussed? **Yes** |
| 23. | Transcripts returned | Were transcripts returned to participants for comment and/or correction? **No** |
| **Domain 3: analysis and findings**z |  |  |
| Data analysis |  |  |
| 24. | Number of data coders | How many data coders coded the data? **One (author TJE)** |
| 25. | Description of the coding tree | Did authors provide a description of the coding tree? **No.** |
| 26. | Derivation of themes | Were themes identified in advance or derived from the data? **Derived from the data** |
| 27. | Software | What software, if applicable, was used to manage the data? **Not applicable.** |
| 28. | Participant checking | Did participants provide feedback on the findings? **No.** |
| Reporting |  |  |
| 29. | Quotations presented | Were participant quotations presented to illustrate the themes / findings? Was each quotation identified? e*.g. participant number* **Yes. Quotation was identified by the participant’s age and gender.** |
| 30. | Data and findings consistent | Was there consistency between the data presented and the findings? **Yes. All findings presented represented the data collected.** |
| 31. | Clarity of major themes | Were major themes clearly presented in the findings? **Yes.** |
| 32. | Clarity of minor themes | Is there a description of diverse cases or discussion of minor themes? **Yes.** |
